# Novel mutations of *COL4A3*, *COL4A4*, and *COL4A5 *genes in Chinese patients with Alport Syndrome using next generation sequence technique

**DOI:** 10.1002/mgg3.653

**Published:** 2019-04-09

**Authors:** Xuechao Zhao, Chen Chen, Yanfu Wei, Ganye Zhao, Lina Liu, Conghui Wang, Junjun Zhang, Xiangdong Kong

**Affiliations:** ^1^ Genetics and Prenatal Diagnosis Center The First Affiliated Hospital of Zhengzhou University, Henan Engineering Research Center for Gene Editing of Human Genetic Disease, Erqi District Zhengzhou China; ^2^ Department of Nephrology The First Affiliated Hospital of Zhengzhou University, Research Institute of Nephrology, Zhengzhou University Zhengzhou China

**Keywords:** Alport syndrome, digenic inheritance, next‐generation sequencing, novel mutations

## Abstract

**Background:**

Alport syndrome (AS) is an inherited progressive renal disease caused by mutations in *COL4A3*, *COL4A4*, and *COL4A5* genes. The large sizes of these genes and the absence of mutation hot spots have complicated mutational analysis by routine PCR‐based approaches. In recent years, the development of next‐generation sequencing (NGS) has made possible the time‐ and cost‐effective and accurate analysis of the three genes in a single step.

**Methods:**

Here, we analyze *COL4A3*, *COL4A4*, and *COL4A5* simultaneously in 29 AS patients using NGS. Candidate mutations were validated by classic Sanger sequencing and Real‐time PCR.

**Results:**

Twenty two new mutations and 10 known mutations were detected. Of those novel mutations, 18, 3, and 1 mutations were detected in *COL4A5*, *COL4A4*, and *COL4A3*, respectively. Twenty six patients showed *X*‐linked inheritance, one showed autosomal recessive inheritance and two showed digenic inheritance (DI).

**Conclusion:**

A comparison of the clinical manifestations caused by different types of mutations in *COL4A5* suggested that large fragment mutations are relatively more severe than the other missense mutations and AS by some mutations may show inter‐ and intra‐familial phenotypic variability. It is important to consider these transmission patterns in the clinical evaluation according to the results of genetic testing, especially for DI. Twenty two new mutations can expand the genotypic spectrum of AS.

## 1 INTRODUCTION

Alport Syndrome (AS) is a clinically and genetically heterogeneous, progressive nephropathy caused by mutations in *COL4A3*, *COL4A4*, and *COL4A5*, which encode type IV collagen (Gubler, 2008; Hudson, Tryggvason, Sundaramoorthy, & Neilson, 2003). The α3, α4 and α5 chains heterotrimer of type IV collagen is essential for the structure and function of the basement membrane in the glomeruli of the kidney, cochlea, and eye. *X*‐linked dominant inheritance pattern (XLAS, OMIM no. 301050) due to mutations in* COL4A5*, which is located in the Xq22 region (Pirson, 1999), accounts for approximately 65% of patients with Alport syndrome; autosomal dominant (ADAS, OMIM no.104200) due to *COL4A3 *or *COL4A4 *heterozygous mutations located in 2q36.3 are related to approximately 20% of patients, and the remaining 15% are autosomal recessive (ARAS, OMIM no. 203780) due to biallelic mutations in* COL4A3* or *COL4A4* (Kashtan, 1993). In addition, with the development of next‐generation sequencing (NGS) technology, the existence of digenic inheritance (DI) has recently been demonstrated in AS with two mutations in the alpha 3‐4‐5 collagen IV genes (Fallerini et al., 2017; Mencarelli et al., 2015).

In AS, a spectrum of phenotypes ranging from progressive renal disease with extrarenal abnormalities (sensorineural deafness and ocular changes) to isolated hematuria with a typically benign course is observed (Wei et al., 2006). The rate of progression to end‐stage renal disease (ESRD) and the presence or absence of sensorineural deafness and ocular changes depends on the mutation they carry (Bekheirnia et al., 2010; Jais et al., 2003). All males with XLAS develop proteinuria and, eventually, progressive renal insufficiency, which leads to ESRD (Barker et al., 1990; Kashtan, 1993). Overall, an estimated 60% reach ESRD by age 30, and 90% by age 40 (Jais et al., 2000). However, the female patients have more variable symptoms, from isolated hematuria to ESRD. Approximately, 12% of females with XLAS develop ESRD before age 40, increasing to 30% by age 60 and 40% by age 80 (Gross, Netzer, Lambrecht, Seibold, & Weber, 2002; Jais et al., 2003). Most individuals with ARAS develop significant proteinuria in late childhood or early adolescence and ESRD before age 30 (Kashtan, 2007; Mochizuki et al., 1994). Progression to ESRD occurs at a slower pace in individuals with ADAS (frequently delayed until later adulthood) than in those with XLAS or ARAS (Kamiyoshi et al., 2016).

AS shows high inter‐ and intrafamilial phenotypic variability, as well as high allelic heterogeneity (Lemmink, Schroder, Monnens, & Smeets, 1997). Approximately, 1,400 different mutations have been collectively reported in the three collagen IV genes (Human Gene Mutation Database, HGMD: http://hgmd.cf.ac.uk, 2018.1). There are 52, 48, and 51 coding exons for *COL4A3*, *COL4A4*, and *COL4A5*, respectively. The large size of these genes and the absence of mutational hot spots have hindered comprehensive genetic screenings in large patient series. In recent years, the development of NGS has made possible the time‐ and cost‐effective and accurate analysis of the three genes in a single step (Artuso et al., 2012; Stokman et al., 2016).

In this study, we applied NGS to analyze* COL4A3*, *COL4A4*, and *COL4A5* simultaneously in 29 patients with clear clinical evidences but no molecular diagnosis.

## 2 MATERIALS AND METHODS

### 2.1 Ethical compliance

The study and procedures were approved by the Research Ethics Committee of Zhengzhou University. All subjects gave their informed signed consents.

### 2.2 Patients and families

According to standard criteria, twenty nine patients diagnosed with AS, were elected from unrelated Chinese families from 75 patients from 2016 to 2017. For each subject, clinical data were collected regarding kidney function (haematuria, proteinuria, chronic renal failure or ESRD) and extra‐renal manifestations (high tone sensorineural hearing loss and ocular lesions). Detailed data on microscopic examination of kidney biopsies were also collected when available. A brief clinical summary of the patients is shown in (Table 1). A sample of peripheral blood in EDTA tubes was collected from probands and all available family members.

**Table 1:** Clinical and pathological features of all the patients

IID	Sex	Age	URBC (10^4^/ml)	U‐P/Cr (g/g)	eGFR (ml/min/1.73 m^2^)	Hearing Loss	EE	Renal biopsy		FH
								**EM**	**α3/α5**	
1	M	21	150	0.45	35	Normal	n.a.	BWC	Nor/A	n.a.
2	M	3	80	1.23	67.8	Normal	n.a.	BWC	M/A	P
3	F	28	650	0.39	80.8	Mild	Normal	BWC	A/A	P
4	M	31	320	0.54	54	Mild	Normal	BWC	M/A	P
5	M	8	258	1.59	61.2	Normal	Normal	BWC	A/A	P
6	M	3	196	1.26	80.9	Normal	n.a.	BWC	A/A	P
7	F	16	78	0.71	112.5	Normal	Normal	BWC	M/A	p
8	M	6	125	0.35	36	Normal	Normal	BWC	M/M	P
9	F	5	101	1.48	68	Normal	Normal	TBM	M/M	P
10	F	2	203	0.97	85.6	Normal	n.a.	TBM	M/M	P
11	F	7	246	0.76	71.7	Normal	Normal	ND	Nor/Nor	P
12	F	11	691	1.27	65.4	Normal	Normal	BWC	M/A	N
13	M	30	450	1.01	20.8	Mild	Normal	BWC	M/A	P
14	M	10	69	1.35	81.3	Normal	Normal	BWC	M/A	P
15	F	33	109	1.46	76.4	Normal	Normal	TBM	M/ M	P
16	M	30	185	0.89	79.9	Mild	Normal	BWC	M/A	P
17	M	8	112	1.59	48.6	Normal	Normal	BWC	Nor/A	P
18	F	9	219	0.91	84.9	Normal	Normal	BWC	Nor/M	P
19	M	1	318	1.06	88.3	Normal	n.a.	BWC	A/A	P
20	M	12	69	0.99	89.2	Normal	Normal	BWC	A/A	P
21	F	5	205	0.41	117.3	Normal	Normal	TBM	Nor/M	P
22	M	15	153	0.54	123.6	Mild	Normal	BWC	M/A	P
23	F	24	94	1.28	92.3	Normal	Normal	BWC	M/ M	n.a.
24	M	24	177	0.89	76.5	Mild	Normal	BWC	M/A	n.a.
25	M	3	82	1.98	41	Normal	n.a.	BWC	Nor/M	P
26	F	4	125	1.87	51.6	Normal	n.a.	BWC	M/ M	P
27	F	37	165	1.12	78.6	Normal	Normal	BWC	Nor/Nor	p
28	F	37	286	1.05	92.9	Normal	Normal	BWC	M/ M	P
29	M	5	376	0.32	76.4	Normal	n.a.	TBM	M/ M	P

Abbreviations: A, absence; BWC, basket‐weave change; EE, eye examination; eGFP, estimated glomerular filtration rate ml/min/1.73 m^2^); EM, electron microscope; FH, family history; IID, individual IDPro, proteinuria (g/24 hr); M, mosatic; N, negative; n.a., not available; Nor, normal; P, positive; TBM, thin basement membrane; U‐P/Cr, urinary protein‐to‐creatinine ratio; URBC, urine red blood cell (10^4^/ml).

### 2.3 Inclusion criteria

All patients diagnosed with AS in this study satisfied one of the following criteria: (a) hematuria and proteinuria or ESRD with renal pathology showing thickening and thinning with lamellation in the glomerular basement membrane (basket weave change [BWC]) and mutations in *COL4A5* or compound heterozygous mutations in *COL4A3* and/or *COL4A4*; (b) hematuria and proteinuria with renal pathology showing thin basement membrane (TBM) and a family history of ESRD, with mutations in *COL4A5* or compound heterozygous mutations in *COL4A3* and/or *COL4A4*.

### 2.4 Samples and DNA extraction

Genomic DNA was extracted from EDTA peripheral blood samples using Lab‐Aid® 824 DNA Extraction Kit according to the manufacturer's protocol (ZEESAN, Xiamen, China).

### 2.5 Custom panel design

In order to perform mutational screening of patients presenting with a clinical suspicion of AS, we created a custom panel for* COL4A3*, *COL4A4, *and *COL4A5* genes using the“Ion AmpliSeq™ designer” software (www.ampliseq.com). We targeted the coding regions and all the flanking introns up to 50 bps. The 3′ and 5′ UTR were not included in the panel design. The total coverage of the panel for the three genes was 99.87%, with 9 bps being left out from *COL4A4*, 87 bps being left out from *COL4A3* and 94 bps from* COL4A5* and it consisted of two different PCR primers pools containing 98 and 96 amplicons respectively, with amplicon sizes ranged from 189 to 238 bps. All missing regions were screened in all samples with Sanger sequencing.

### 2.6 Ion torrent PGM sequencing

The library preparation was performed by amplifying 10 ng of genomic DNA, using the Ion AmpliSeq™ Library Kit 2.0 (Life Technologies). This kit allowed obtaining a barcoded library of the 194 amplicons, corresponding to the 151 exons of *COL4A3/COL4A4/COL4A5* genes compatible with the Ion PGM platform, according to the Life Technologies protocol. Libraries were purified using Agencourt^®^ AMPure^®^ XP system and quantified using the Qubit^®^ dsDNA HS Assay Kit reagent (Invitrogen Corporation, Life Technologies, Carlsbad, CA), pooled at an equimolar ratio, annealed to carrier spheres (Ion Sphere™ Particles, Life Technologies) and clonally amplified by emulsion PCR (emPCR) using the Ion OneTouch™ 2 system (Ion PGM™ Template Hi‐Q™ view OT2 200 kit, Life Technologies). The spheres, carrying single‐stranded DNA templates, were loaded to 316™v2 chip and sequenced on the Ion Torrent PGM, using the Ion PGM™ Hi‐Q™ view Sequencing 200 kit v2, according to the protocol of Life Technologies. Postrun analysis was conducted using the latest version (v5.0.4) of the data analysis software Torrent Suite™ (Life Technologies). Coverage assessment was performed using the “coverage Analysis” plug‐in (v5.0.4) that gives information about the amplicons read coverage and variants were called using the “variant Caller” plug‐in (5.0.4).

### 2.7 Real‐time PCR

To identify the fragment deletion, real‐time PCR was performed using a SYBR Green PCR kit (TAKARA) in a Real‐Time PCR Detection System (BIO‐RAD IQ2). Melting curve analysis was performed to ensure the amplification of a single product. The data were analyzed according to the comparative Ct method. Relative quantification (RQ) = 2^‐∆∆Ct^.

## 3 RESULTS

### 3.1 Identification of candidate mutations in *COL4A3*, *COL4A4*, and *COL4A5*

In the 29 patients, we identified 20 missense mutations, two nonsense mutations, three frameshift mutations, two deletion mutations, and five splicing site mutations. Among these, 10 were known mutations that had been reported previously and 22 were novel ones (Table 2); 50% (16/32) were new amino acid substitutions of glycine. These candidate mutations were validated by Sanger sequencing. The predicted clinical significances of these mutations are listed in Table 2 and the criteria of clinical significance are based on American College of Medical Genetics (Richards et al., 2015).

**Table 2:** Mutations detected in *COL4A3*, *COL4A4*, and *COL4A5*

Sample IID	Gene Symbol	Zygosity	Function	cHGVS	pHGVS	Clinical significance	Fr.1	Fr.2	Fr.3	Comment
1	COL4A5	hem	Missense	c.3799G > A	p.(Gly1267Ser)	VUS	–	–	–	Novel
2	COL4A5	hem	Missense	c.1717G > A	p.(Gly573Ser)	VUS	–	–	–	Novel
3	COL4A5	het	Missense	c.4550G > A	p.(Arg1517His)	LP	–	0.00002	–	Known (Cheong, Park, Ha, & Choi, 2000)
		het	Nonsense	c.4705C > T	p.(Arg1569Ter)	P	–	–		Known (Zhou et al., 1993)
4	COL4A5	hem	Missense	c.2210G > A	p.(Gly737Asp)	VUS	–	–	–	Novel
5	COL4A5	hem	Deletion	del ex37−46	–	P	–	–	–	Novel
6	COL4A5	hem	Deletion	del ex35−36	–	P	–	–	–	Novel
7	COL4A5	het	Frameshift	c.3306_3313del	p.(Pro1103Alafs*31)	P	–	–	–	Novel
8	COL4A5	hem	Missense	c.2723G > A	p.(Gly908Glu)	VUS	–	–	–	Novel
9	COL4A5	het	Frameshift	c.1151delT	p.(Pro385Leufs *89)	P	–	–	–	Novel
10	COL4A5	het	Missense	c.4175T > C	p.(Leu1392Pro)	VUS	–	0.00003	0.000529	Novel
11	COL4A5	het	Missense	c.1754G > C	p.(Gly585Ala)	VUS	–	–	–	Novel
12	COL4A5	het	Missense	c.1807G > A	p.(Gly603Ser)	VUS	–	–	–	Novel
13	COL4A5	hem	Missense	c.2597G > A	p.(Gly866Glu)	LP	–	–	–	Known (Renieri et al., 1996)
14	COL4A5	het	Frameshift	c.3706delC	p.(Pro1237Qlnfs*68)	P	–	–	–	Novel
15	COL4A5	het	Missense	c.973G > A	p.(Gly325Arg)	LP	–	–	–	Known (Knebelmann et al., 1992)
16	COL4A5	hem	Missense	c.4462G > C	p.(Gly1487Ala)	LP	–	–	–	Known (Plant, Green, Vetrie, & Flinter, 1999)
17	COL4A5	hem	Missense	c.1957G > A	p.(Gly653Arg)	LP	–	–	–	Known (Boye et al., 1995)
18	COL4A5	het	Missense	c.2215C > G	p.(Pro739Ala)	VUS	0.0028	0.0034	0.011416	Known (F. Wang et al., 2012)
19	COL4A5	hem	Missense	c.3535G > A	p.(Gly1179Arg)	LP	–	–	0	Known (Nagel, Nagorka, & Gross, 2005)
20	COL4A5	hem	Missense	c.2632G > A	p.(Gly878Arg)	VUS	–	–	–	Novel
21	COL4A5	het	Missense	c.688G > A	p.(Gly230Ser)	VUS	–	–	–	Novel
22	COL4A5	hem	Missense	c.1616G > T	p.(Gly539Val)	VUS	–	–	–	Novel
23	COL4A5	het	Missense	c.2351G > T	p.(Gly784Val)	VUS	–	–	–	Novel
24	COL4A5	hem	Splicing	c.277‐1G > T	–	P	–	–	0	Known (Plant et al., 1999)
25	COL4A5	hem	Splicing	c.439‐1G > A	–	P	–	–	–	Known (Hanson, Storey, Pagan, & Flinter, 2011)
26	COL4A5	het	Splicing	c.2395 + 2T>C	–	P	–	–	–	Novel
27	COL4A5	het	Splicing	c.1339 + 3A>T	–	VUS	–	–	–	Novel
	COL4A4	het	Missense	c.4421C > T	p.(Thr1474Met)	VUS	0.0004–	0.0001	0.00015	Novel
28	COL4A4	het	Missense	c.4421C > T	p.(Thr1474Met)	VUS	0.0004–	0.0001	0.00015	Novel
		het	Splicing	c.694‐2A > C	–	P	–	–	–	Novel
29	COL4A3	het	Missense	c.3356G > A	p.(Gly1119Asp)	VUS	–	0.000008	0.000012	Novel
	COL4A4	het	Nonsense	c.5026C > T	p.(Gln1676Ter)	LP	–	–	–	Novel

Note

IID, individual ID, het, heterozygous, hem, hemizygous, LP, likely Pathogenic, p, pathogenic and VUS, variant uncertain significance.

*COL4A3* reference transcript NM_000091.4; *COL4A4* reference transcript NM_000092.4; *COL4A5* reference transcript NM_033380.

Fr.1: Frequency in 1,000 genome database

Fr.2: Frequency in ExAC

Fr.3: Frequency in CNGMD (Chinese Gene Mutation Database)

The criteria of clinical significance are based on American College of Medical Genetics.

**Figure 1:**
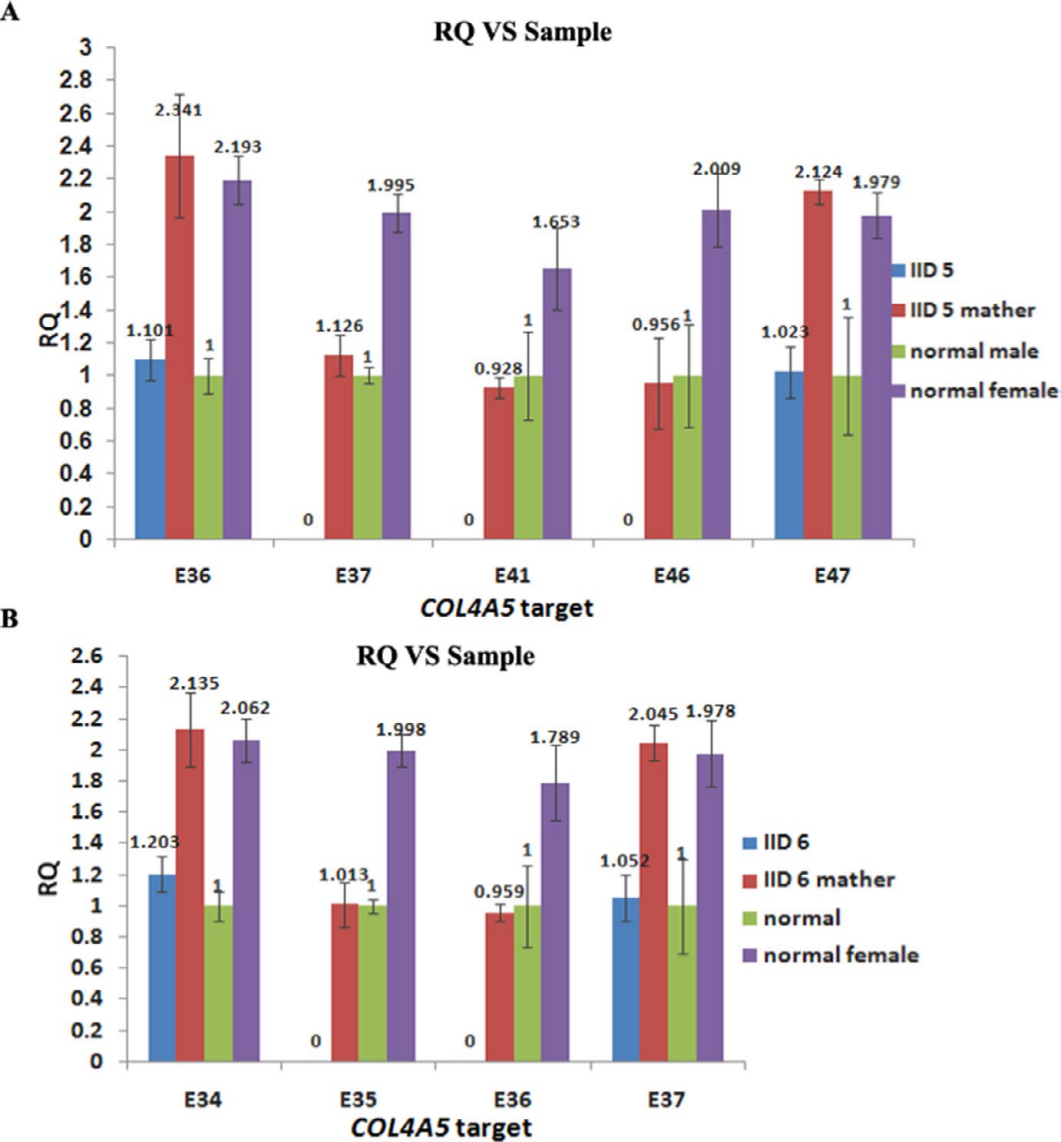
The identification of fragment deletions in *COL4A5*. (a) The PCR quantification results of IID5. The comparison of quantification of exons 37–46 of *COL4A5* between patient IID5 and controls. (b) The PCR quantification results of IID6. The comparison of quantification of exons 35–36 of *COL4A5* between patient IID6 and controls. RQ, real‐time quantitative PCR

There are two patients with AS carrying two mutations in two distinct collagen IV genes. In AS patient IID27, the two mutations in *COL4A5* and *COL4A4* were inherited independently, likely indicating an *in trans* configuration. There is a splicing site mutation c.1339 + 3A>T in *COL4A5, *inherited from her mother and a missense mutation c.4421C > T (p. (Thr1474Met)) inherited from her father (Figure 1a). In AS patient IID29, in addition to a glycine substitution (p. (Gly1119Asp)) in *COL4A3 *in the heterozygous state, there was another heterozygous nonsense mutation c.5026C > T in *COL4A4* genes. The two mutations were *in cis* configuration, inherited together on the same chromosome from her father (Figure 1b).

### 3.2 Identification of fragment deletions in *COL4A5*

**Figure 2:**
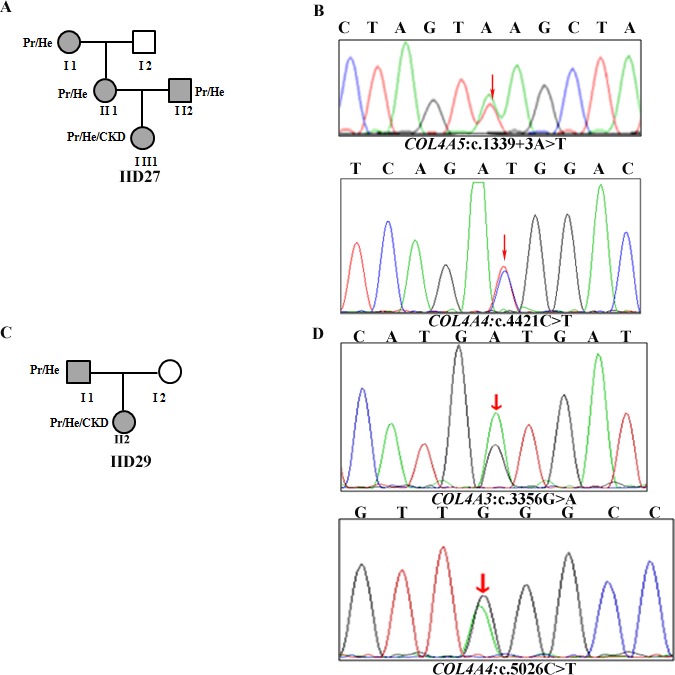
(a) and (c): The pedigrees of the Alport syndrome (AS) family with putative digenic inheritance. (b) and (d): The new variants and flanking sequences are shown. Pr: proteinuria, He: hematuria, CKD: chronic kidney disease

There are two patients carrying fragment deletion in *COL4A5*, which were validated by qPCR (Figure 2). A hemizygous deletion of exon 37–46 was identified in IID5 (Figure 2a), who had hematuria and proteinuria at age 8, and the deletion was inherited from his mother, who had hematuria. In IID6, who had intermittent hematuria and proteinuria since age 2, there was a hemizygous deletion of exon35‐36 (Figure 2b) and the deletion was inherited from his mother, who had hematuria and proteinuria.

## 4 DISCUSSION

Of the 29 AS patients, 26 showed *X*‐linked inheritance (mutations in *COL4A5*), one showed autosomal recessive inheritance, and two showed DI. Among 32 mutations, 16 (50%) were new amino acid substitutions of glycine. Indeed, glycine substitutions within the repetitive triplet sequence (Gly)‐*X*‐*Y* of the collagenous domain represent one of the most common type of pathogenic variant found in AS patients (Liu et al., 2017), as they are suspected to introduce kinks in the molecule, thus interfering with the proper folding of the collagen triple helix (Kashtan, 1993; Wang, Ding, Wang, & Bu, 2004). Overall, symptoms of female in XLAS exhibited more modest clinical manifestations than male in XLAS; patients with nonsense mutations, frameshift mutations, deletion mutations and splicing mutations presented relatively more severe symptoms than others. Female patients IID7 (p. (Pro1103Alafs*31)), IID9 (p.(Pro385Leufs *89)), and II26 (c.2395 + 2T>C) exhibited only moderate proteinuria, microscopic hematuria, and normal renal function. Male patients IID27 (c.2146 + 2T>A) and IID5 (deletion of exon37‐46) presented relatively more severe symptoms, such as grosser hematuria or proteinuria and chronic kidney disease (CKD) and IID24 showed grosser hematuria, proteinuria and extra renal manifestations (Table 1). Patients IID6 (deletion of exon35‐36) and IID25 (c.439‐1G > A) exhibited the modest clinical symptoms, but considering their young age, further follow‐up is necessary for their clinical symptoms.

IID3 with compound heterozygosity mutations (p. (Arg1517His) and p. (Arg1569Ter)) in* COL4A5* inherited from her parents showed grosser hematuria, proteinuria, CKD and with extra renal manifestations. Although the two mutations have been reported as pathogenic, her mother with p.(Arg1569Ter) exhibited similar severe symptoms to the proband and her son (6 years)with p.(Arg1569Ter) presented hematuria and proteinuria; however, her father with p.(Arg1517His) did not presented any symptoms related to AS. This may be attributed to inter‐familial phenotypic variability.

The patient IID28 with acute tubular injury carried two mutations (p. (Thr1474Met) from mother in *COL4A4 *and c.694‐2A > C from father in *COL4A4*) and showed autosomal recessive inheritance. Her father (69 years) exhibited hematuria, proteinuria and extra renal manifestations and was diagnosed with ADAS. Her mother (67 years) exhibited hematuria and proteinuria. This may indicate that splicing mutation c.694‐2A > C probably played a dominant role in patient IID28.

In this study, using NGS (Ion Torrent PGM platform), we identified two AS families harboring mutations in two distinct collagen IV genes (digenic model) (Table 2). In patient IID27, we detected a de novo mutation in *COL4A5* combined with a *COL4A4 *mutation. The female patient presents a severe phenotype, showing hematuria, proteinuria, and CKD. Her mother with c.1339 + 3A>T in *COL4A5* and her father with a missense mutation c.4421C > T in *COL4A4 *had intermittent hematuria and proteinuria. In proband of family 29, in addition to a glycine substitution (p. (Gly1119Ala)) in *COL4A3 *in the heterozygous state, there was another heterozygous nonsense mutation c.5026C > T in *COL4A4* genes. The two mutations were *in cis* configuration, inherited together on the same chromosome from her father. The proband exhibited hematuria, proteinuria, and renal insufficiency and her father showed microhematuria and proteinuria. This may be attributed to intrafamilial phenotypic variability. In this case, the inheritance pattern mimics an autosomal dominant form with a recurrence risk of 50%. However, the phenotype is more severe compared to an autosomal dominant pattern (Fallerini et al., 2017). This may indicate that AS patients with mutations in two different collagen genes show a more severe phenotype compared with those with a single mutation (Liu et al., 2017). According to the stoichiometry of the molecules of the triple helix, in double heterozygotes, about 75% of triple helix molecules are expected to be defective, which is > 50% in heterozygotes and < 100% in homozygotes or hemizygotes (Mencarelli et al., 2015). Therefore, in the case with mutations on the same autosomal chromosome (*in cis*) (family 30), a clinical re‐evaluation on the basis of molecular data highlights the importance of considering a worse prognosis in comparison with an autosomal dominant mode of inheritance.(Artuso et al., 2012; Fallerini et al., 2014; Mencarelli et al., 2015; Pescucci et al., 2004). Conversely, a better prognosis should be considered in comparison with an autosomal recessive mode of inheritance, if the two mutations are independently inherited (*in trans*) (Fallerini et al., 2017; Wang et al., 2014; Zhang et al., 2012).

We analyzed the frequencies of the identified variant c. 2215C > G in *COL4A5 *from IID18 in ExAC (0.28%), 1,000 genome (0.34%) database and Chinese Gene Mutation Database (http://cngmd.virgilbio.com) (1.14% (more low in Han people (0.93%)). But more than 5% in the Japanese healthy population have this variant in *COL4A5* (http://www.hgvd.genome.med.kyoto-u.ac.jp/cgi-bin/frequency_plot.cgi?chr=chrX&range=107846262-107846262-4&org_xml:id=1). The result may imply that there are some differences at some variants among different populations. The proband was a 9‐year‐old female with first onset of gross hematuria at the age of 6. By the age of 7 years, she had already developed proteinuria, and laboratory investigation also confirmed high plasma creatinine. His mother showed intermittent microhematuria and proteinuria, and progressed to chronic kidney disease. Her grandmother and two sisiters exhibited hematuria and proteinuria. This mutation was shown to co‐segregate with the AS phenotype in the family. In addition, it has been reported that the c.2215C > G mutation may be one of pathogenic mutations underlying FSGS in Chinese (Zhang, Yang, & Hu, 2017). Therefore, we think that it was worth to discuss the clinical significance of this variant in Chinese.

In fact, we also found eight patients carrying a heterozygosity mutation in *COL4A4 *or *COL4A3*. These probands showed hematuria, low dose of proteinuria, and TBM, but was absence of a family history of ESRD (data not shown). Considering the modest clinical symptoms and their young age, further follow‐up is necessary for their clinical symptoms (Mochizuki et al., 1994). This will be attributed to diagnosis these probands as ADAS or thin basement membrane nephropathy, which is also related to mutations in collagen IV genes (Stokman et al., 2016).

In conclusion, genetic testing is of great and increasing importance for diagnosing AS. The development of NGS has made possible the time‐ and cost‐effective and accurate analysis of the three genes in a single step (Artuso et al., 2012; Moriniere et al., 2014). In this study, we think it is important to consider these transmission patterns in the clinical evaluation according to the results of genetic testing. The diversity of inheritance pattern in *COL4A3*,* COL4A4, *and* COL4A5 *can help us to explain the variable clinical expression of the disease. The identification of transmission patterns by NGS permit us to consider the recurrence risk in the families and make more reasonable genetic and prenatal counseling for AS.

## CONFLICT OF INTEREST

The authors report no relevant conflicts of interests related to the manuscript.
